# Supplementary material for: Quantum magnetic J-oscillators
Source: Nat Commun. 2026 Jan 29;17:1200. doi: 10.1038/s41467-026-68779-5 (PMC12859129; doi:10.1038/s41467-026-68779-5)
Supplement: Supplementary file 1 — Supplementary Information [file 41467_2026_68779_MOESM1_ESM.pdf]

Supplementary Information
Quantum Magnetic J-Oscillators

Jingyan Xu[1,2,3], **Raphael Kircher**[1,2,3], **Oleg Tretiak**[1,2,3], **Dmitry Budker**[1,2,3,4], and **Danila A. Barskiy**[1,2,3,5,*]

[1]*Helmholtz Institute Mainz, Mainz 55128, Germany*
[2]*GSI Helmholtzzentrum für Schwerionenforschung, Darmstadt 64291, Germany*
[3]*Institute of Physics, Johannes Gutenberg-Universität, Mainz 55099, Germany*
[4]*Department of Physics, University of California, Berkeley, CA 94720, USA*
[5]*Frost Institute for Chemistry and Molecular Science, Department of Chemistry, University of Miami, Coral Gables, FL 33146, USA*

[*]Corresponding author: `barskiy@miami.edu`

Supplementary methods—Parahydrogen handling

Hydrogen gas is converted to *para*-H_2 by passing commercially available di-hydrogen gas over a packed bed of iron oxide catalyst FeO(OH) at a temperature of 30 K. This process yielded a *para*-H_2 enrichment of approximately 97%. After conversion, the gas is transported through a piping system composed primarily of polyetheretherketone (PEEK). The gas is warmed to approximately 21 °C and pressurized to 10 bar before it is bubbled into the sample.

The liquid sample is contained in a custom glass NMR tube consisting of a 5 mm upper cylindrical neck fused to a spherical bottom bulb with an inner diameter of 14.5 mm. Two Teflon capillaries are inserted into the tube: an inlet capillary positioned at the bottom of the sample volume for gas bubbling, and an outlet capillary positioned in the headspace above the liquid. The internal pressure of the NMR tube is maintained at 7 bar using a back-pressure regulator (Swagelok). Gas flow into the tube is regulated at 20 sccm (standard cubic centimeters per minute) using a mass flow controller (Sierra Instruments). This low flow rate was selected to minimize solvent evaporation.

For J-oscillator measurements, spectra are collected under continuous *para*-H_2 bubbling. In conventional zero-field NMR experiments, hyperpolarization was achieved via a intermittent bubbling sequence consisting of five repetitions. Each cycle involves 2.6 s of bubbling followed by a 0.5 s delay. The magnetic field sequence used is the "ZF-ZF" sequence, where the sample is hyperpolarized at zero field via SABRE, then excited by a non-adiabatic DC pulse (40 μT for 133 μs) prior to signal detection at zero field [1]. All timing sequences, pneumatic valve actuation, and hardware communication were automated using TTL pulses controlled via a custom Python script.

Supplementary Notes 1—The detailed theory of J-oscillator

The explicit forms of the operators and superoperators introduced in,

$$\frac{d}{dt}\hat{\rho}(t) = -i[\hat{H}_0 + \hat{V}(t), \hat{\rho}(t)] + \hat{\hat{R}}\hat{\rho}(t) + \hat{P}, \tag{S1}$$

which govern the dynamics of the J-oscillations, are detailed below for the representative case of [^{15}N]-acetonitrile.

Hamiltonian

The J-coupling interaction Hamiltonian for the [^{15}N]-acetonitrile molecular system is:

$$\hat{H}_0 = 2\pi J(\hat{\mathbf{S}} \cdot \hat{\mathbf{K}}), \tag{S2}$$

where $\hat{\mathbf{S}}$ and $\hat{\mathbf{K}}$ are the spin operators for ^{15}N and total ^{1}H nuclei, respectively, and J is the scalar coupling constant. The interaction with the feedback magnetic field is modeled as:

$$\hat{V}(t) = -G_{\text{ext}} \cdot B_{\text{OPM}}(t - \tau) \cdot (\gamma_{^{15}\text{N}} \hat{S}_{\text{y}} + \gamma_{^1\text{H}} \hat{K}_{\text{y}}), \tag{S3}$$

where G_{ext} is the (external) feedback gain, τ is externally applied the feedback delay, and $\gamma_{^{15}\text{N}}$, $\gamma_{^1\text{H}}$ are gyromagnetic ratios of ^{15}N and ^{1}H nuclei, respectively. Consequently, we define $B_{\text{ext}} = G_{\text{ext}} \cdot B_{\text{OPM}}$. The field from the spherically shaped sample as measured by the OPM, $B_{\text{OPM}}(t)$ can be derived from the sample magnetization $M_y(t)$:

$$B_{\text{OPM}}(t) = -\frac{\mu_0}{3}\frac{r^3}{d^3} M_y(t). \tag{S4}$$

Here, $d = 12.45\,\text{mm}$ is the distance between the center of the sensor cell and the sample, and $r = 4.2\,\text{mm}$ is the radius of the sample. The magnetization of the system $M(t)$ (would be along the y-axis due to the uniaxial nature of zero-field scalar maser) is calculated as,

$$M_y(t) = \langle \gamma_{^{15}\text{N}} \hat{S}_{\text{y}} + \gamma_{^1\text{H}} \hat{K}_{\text{y}} \rangle (t) \cdot \hbar \cdot N_{\text{A}} \cdot C, \tag{S5}$$

where $C = 967\,\text{mM}$ is the [^{15}N]-ACN concentration, and N_{A} is the Avogadro's constant. The notation $\langle \cdot \rangle = \text{Tr}(\hat{\rho}(\text{t}) \cdot)$ denotes the quantum expectation value.

Relaxation

The relaxation superoperator incorporates intramolecular dipolar interactions, intermolecular interactions, and paramagnetic effects. Intramolecular relaxation is modeled using a rotational diffusion approach for a symmetric top, with tunneling and spinning diffusion times of 0.135 ps and 3 ps, respectively. Details of the calculation method will be given elsewhere.

Intermolecular effects and paramagnetic contributions (e.g., dissolved oxygen) are treated via a random fluctuating field model [2]:

$$\hat{H}_{\text{fluc}}(t) = -\sum_{j=x,y,z}\left(\gamma_{^{15}\text{N}}\, B_{Sj}(t)\, \hat{S}_j + \gamma_{^1\text{H}}\, B_{Kj}(t)\, \hat{K}_j\right) \tag{S6}$$

where $B_{Sj}(t)$ and $B_{Kj}(t)$ are random fields along orthogonal axes. Their correlations satisfy:

$$\frac{1}{2}\int_{-\infty}^{\infty} dt'\, \gamma_{^1\text{H}}^2 \overline{B_{Ij}(t) B_{I'j'}(t-t')} = \frac{1}{T_s}\delta_{jj'} C_{II'}\,, \tag{S7}$$

with $C_{II'} = 1$ for $I = I'$ and 2/3 otherwise based on the assumption that the noise field applied on ^{15}N and ^{1}H spins are partially correlated. The calculation of the corresponding relaxation superoperator is based on the work [3].

Chemical exchange effects from SABRE were found negligible, as evidenced by minimal linewidth differences in J-spectra with different catalyst concentrations as shown in Supplementary Fig. 15.

SABRE-pumping

The pumping term $\hat{P}$ ensures steady-state state aligns with the SABRE-hyperpolarized population imbalances at zero-field,

$$\hat{\hat{R}}\hat{\rho}_{\text{eq}} + \hat{P} = 0\,. \tag{S8}$$

In the absence of external field, the steady state is isotropic, i.e., it has no preferred directions in space. As a result, the quantum states with the same total angular momentum (F) and total proton angular momentum (K) have the same populations. And the steady-state $\hat{\rho}_{\text{eq}}$ can be fully describe by two parameters (see Supplementary Fig. 16), α (for population imbalances in $K = 3/2$ manifold) and β (for population imbalance in $K = 1/2$ manifold). The values of are determined from the integrated signals of 1-J (I_{1J}) and 2-J peaks (I_{2J}) from a "ZF-ZF" experiment [1] with a 90° ^{15}N-^{1}H DC pulse:

$$\alpha = \frac{I_{1J}}{8b_0}\,,\ \ \beta = \frac{I_{2J}}{20b_0} \tag{S9}$$

with,

$$b_0 = \frac{1}{2}(\mu_{^1\text{H}} - \mu_{^{15}\text{N}}) \cdot C \cdot N_{\text{A}} \cdot \frac{\mu_0}{3} \cdot \frac{r^3}{d^3}\,. \tag{S10}$$

Here, $\mu_{^1\mathrm{H}}$ and $\mu_{^{15}\mathrm{N}}$ are the magnetic dipole moment for ^{15}N and ^{1}H nucleus, respectively.

The numerical solver

The master equation is solved via Strang splitting [4], which separates coherent and dissipative dynamics with second-order accuracy. The density operator $\hat{\rho}(t)$ evolves over a timestep Δt as follows:

1. **Coherent half-step:** Apply $\hat{U}_1 = \exp\Big[-i\big(\hat{H}_0 + \hat{V}(t+\frac{\Delta t}{4})\big)\frac{\Delta t}{2}\Big]$, updating $\hat{\rho} \to \hat{U}_1\,\hat{\rho}\,\hat{U}_1^\dagger$.
2. **Dissipative step:** Update $\hat{\rho}$ via an Euler step, $\hat{\rho} \to \hat{\rho} + \big(\hat{\hat{R}}\hat{\rho} + \hat{P}\big)\Delta t$.
3. **Second coherent half-step:** Apply $\hat{U}_2 = \exp\Big[-i\big(\hat{H}_0 + \hat{V}(t+\frac{3\Delta t}{4})\big)\frac{\Delta t}{2}\Big]$, updating $\hat{\rho} \to \hat{U}_2\,\hat{\rho}\,\hat{U}_2^\dagger$.

Simulation Parameters

The model includes three key parameters: I_{1J}, I_{2J} and T_s. For Fig. 3C-3D, we used $I_{1J} = 58\,\mathrm{pT}$, $I_{2J} = 106\,\mathrm{pT}$ and $T_s = 28\mathrm{s}$. For Fig. 3E-3F, these parameters were $I_{1J} = 66\,\mathrm{pT}$, $I_{2J} = 124\,\mathrm{pT}$ and $T_s = 32\,\mathrm{s}$. Note the difference between the two parameter sets arise because the samples were prepared on different days.

Supplementary Notes 2—The normalization of Fourier Transformation

Suppose a continuous signal $x(t)$ is sampled at a rate f_s to get a discrete sequence

$$x[n] = x\Big(t = \frac{n}{f_s}\Big), \quad n = 0, 1, \ldots, N-1, \tag{S11}$$

over a total duration $T = N/f_s$. The discrete Fourier transform (DFT) is then calculated as,

$$X[k] = \sum_{n=0}^{N-1} x[n]\, e^{-i\frac{2\pi k}{N}n}, \quad k = 0, 1, \ldots, N-1. \tag{S12}$$

Interpreting the sum as a Riemann approximation, i.e., $\Delta t \cdot \sum(\cdot) \approx \int_0^T (\cdot)\, dt$, $X[k]$ approximates the continuous Fourier integral,

$$X[k] \approx f_s \int_0^T x(t)\, e^{-i2\pi f_k t}\, dt, \tag{S13}$$

with the discrete frequency $f_k = \frac{kf_s}{N}$ and the sampling interval $\Delta t = 1/f_s$.

The two different normalization convention adopted in the main work are:

Convention I: Divide $X[k]$ by the number of samples N, giving,

$$\widetilde{X}_{\mathrm{I}}[k] = \frac{X[k]}{N} \approx \frac{1}{T} \int_0^T x(t)\, e^{-i2\pi f_k t}\, dt. \tag{S14}$$

In this case, the Fourier amplitudes retain the original units of $x(t)$ (e.g., pT). This convention is adopted in Fig. 4 and Fig. 5.

Convention II: Multiply $X[k]$ by $\Delta t = 1/f_s$, so that,

$$\widetilde{X}_{\mathrm{II}}[k] = \frac{X[k]}{f_s} \approx \int_0^T x(t)\, e^{-i2\pi f_k t}\, dt. \tag{S15}$$

This yields Fourier amplitudes with units such as pT/Hz (if $x(t)$ is measured in pT). This convention is adopted in Fig. 1 and Fig. 2.

Supplementary Figures

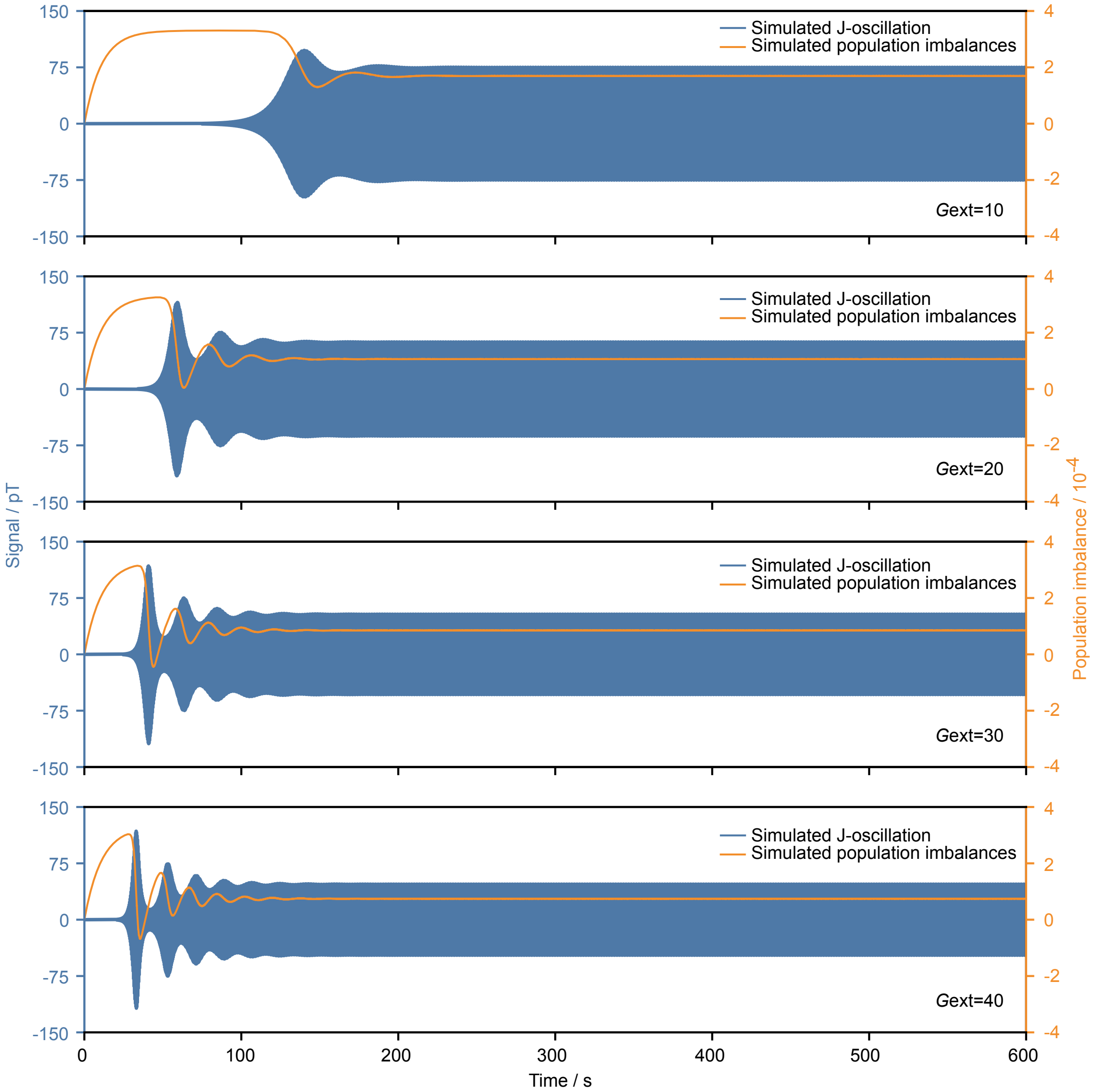

Supplementary Figure 1: Simulations of J-oscillators as a function of the feedback gain. The blue trace represents the oscillator signal while the orange trace shows the population difference, calculated as (3/4) × (population in $K = 3/2$, $F = 2$ states) - (5/4) × (population in $K = 3/2$, $F = 1$ states); the scaling factors (3/4 and 5/4) account for the different number of available states in $F = 2$ versus $F = 1$ (see Supplementary Figure 16). As the feedback gain increases, the initial SABRE-pumped population imbalance can be inverted during the burst events. The simulations follow the Methods section, with the feedback delay fixed to $\tau = 222\,\mathrm{ms}$ and the rms of the OPM noise set to 0.1 pT.

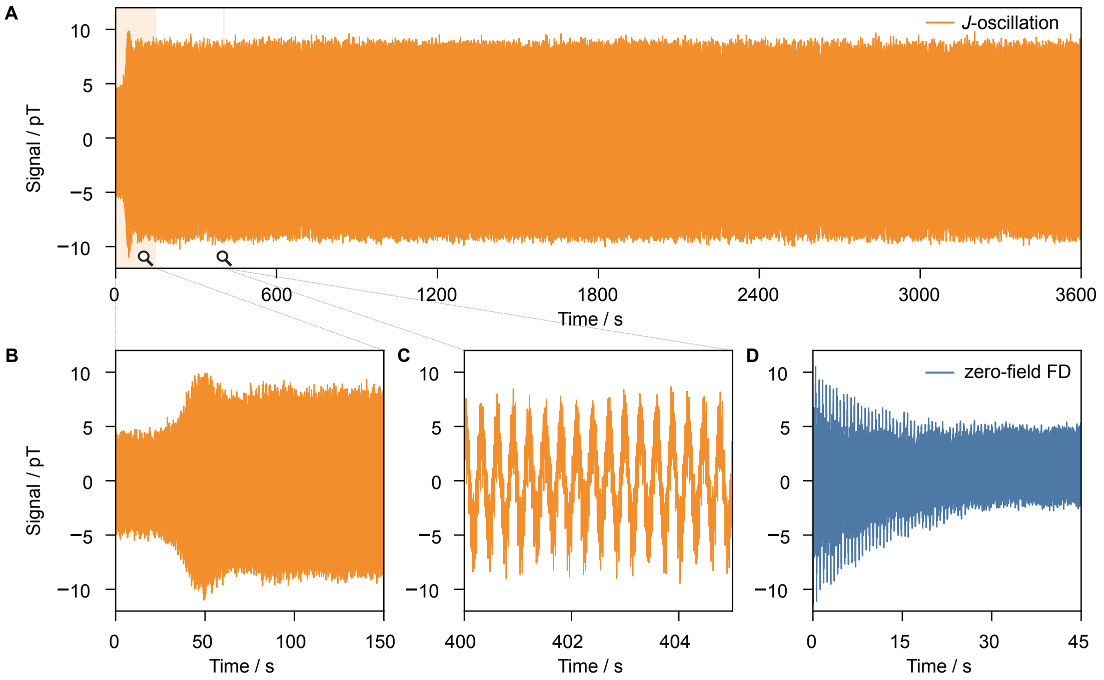

Supplementary Figure 2: **Time-domain signal measured from neat acetonitrile solvent at natural isotopic abundance.** (A) Experimentally recorded quantum J-oscillator time-domain signal obtained with $\tau = 222\,\text{ms}$ and $G_{\text{ext}} = +200$. (B) Zoom-in of panel A showing the spontaneous emergence of the J-oscillation. (C) Zoom-in of panel A showing the dynamic steady-state oscillation. (D) Zero-field free decay (FD) signal of the same sample for reference. This figure illustrates the ability to create and sustain J-oscillators on readily available solvents at natural isotopic abundance.

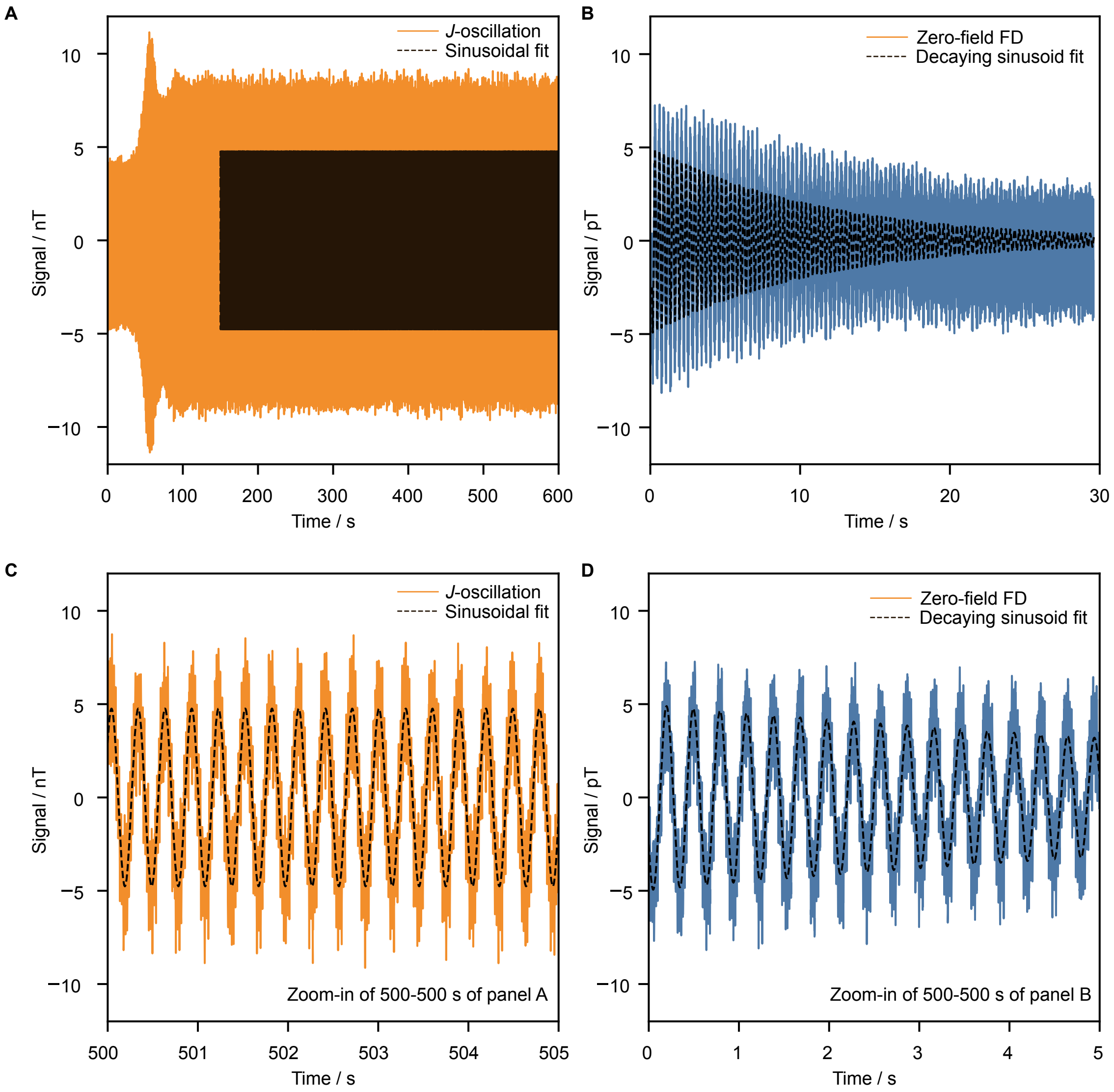

Supplementary Figure 3: Example of frequency extraction from time-domain signals. (A) Time-domain trace of the J-oscillator signal with black lines representing sinusoidal fits used for frequency extraction. (B) Time-domain trace of the free-decay (FD) signal with black lines showing decaying sinusoidal fits. (C) Zoomed-in section of panel A. (D) Zoomed-in section of panel B.

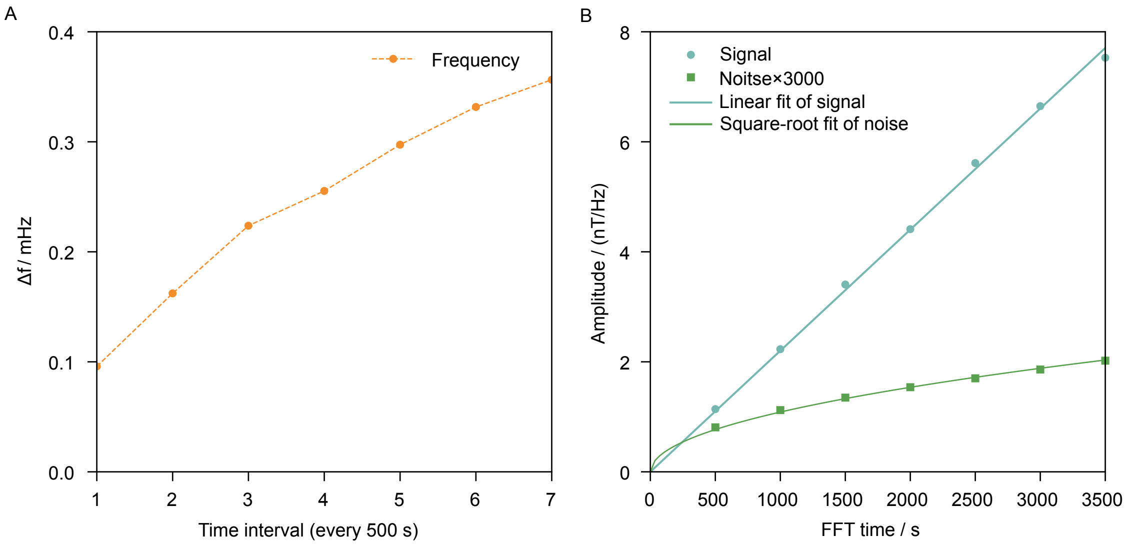

Supplementary Figure 4: Frequency and spectral characteristics of the quantum oscillator derived from the time-domain signal in Supplementary Figure 2. (A) J-oscillation frequency extracted from successive 500 s segments by sinusoidal fitting (Δf is referenced to 3.374 Hz). (B) Signal amplitude of FFT spectra obtained from the signal using sliding windows starting at 100 s with increasing durations. The noise floor was evaluated over the 8–10 Hz range. Solid lines are fits to the data using a linear function for the spectral amplitude and a square-root function for the noise floor.

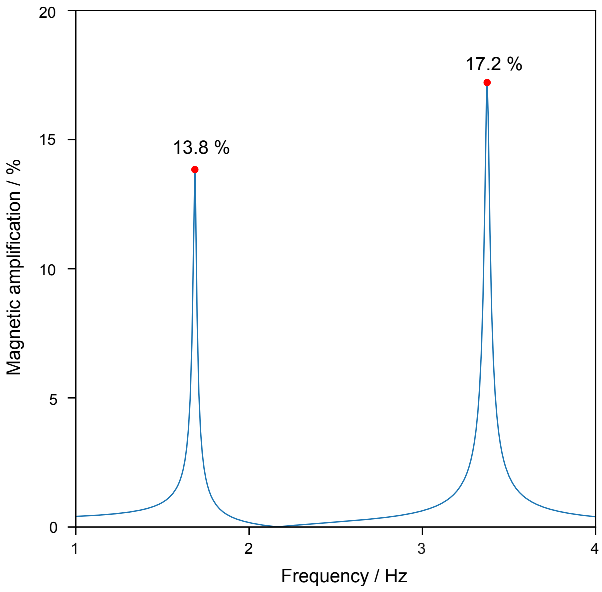

Supplementary Figure 5: Simulated dependence of the intrinsic magnetic amplification (G_{int}) of the system on the frequency (f) of AC excitation. The simulations show maximal amplification at $f = J$ and $f = 2J$ ($J = 1.687\,\text{Hz}$), with internal gains $G_{\text{int}}(J) \approx 13.8\,\%$ and $G_{\text{int}}(2J) \approx 17.2\,\%$, respectively. The simulations follow the Methods section, with the same parameters as used for the simulations in Fig. 3E-3F.

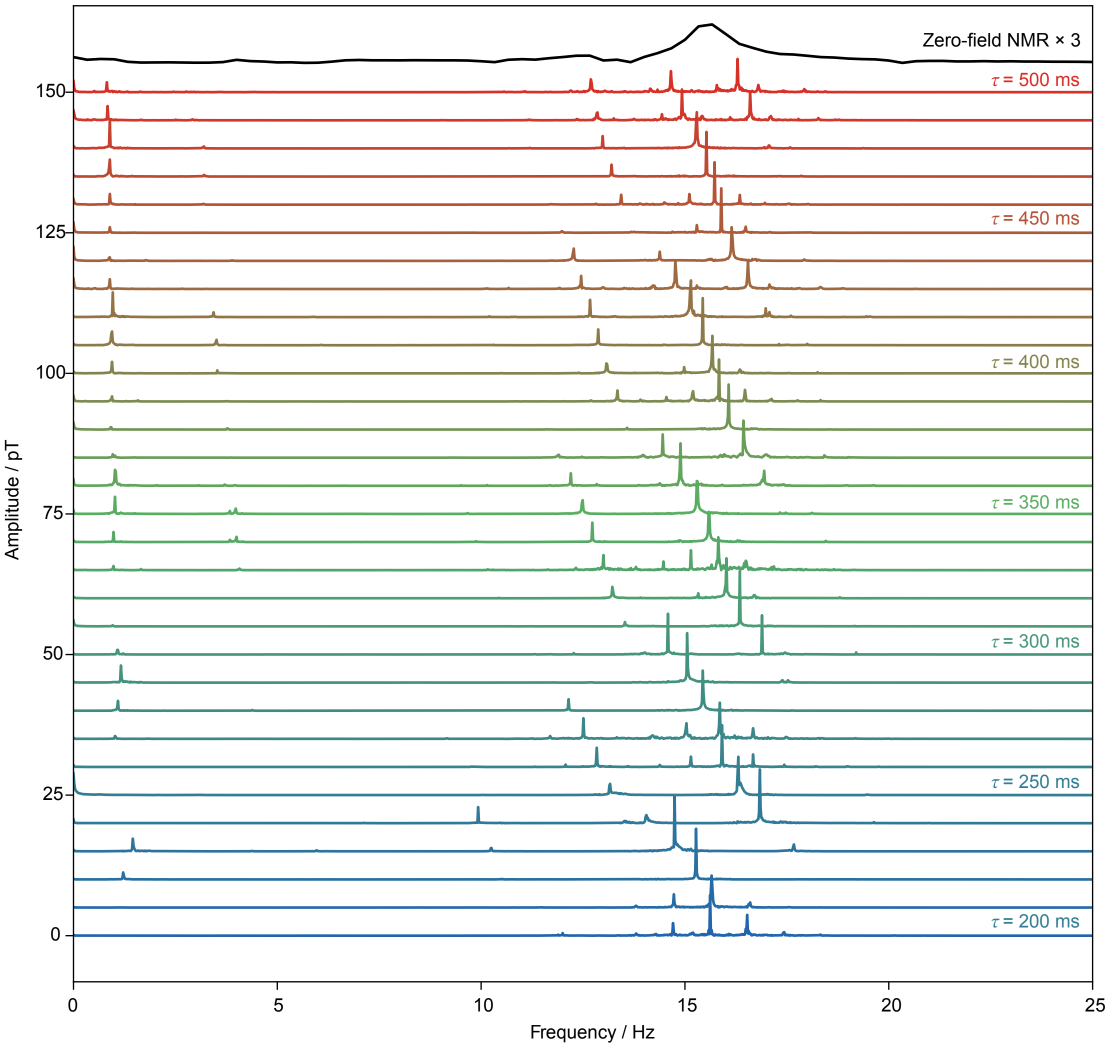

Supplementary Figure 6: **Stacked spectra from quantum oscillators in [^{15}N]-pyridine acquired at varying feedback delays (τ).** The external feedback gain was fixed at $G_{\mathrm{ext}} = -3000$ for all experiments. Each spectrum corresponds to a 1 min acquisition, with Fourier transformation applied to the time-domain data from 5–60 s to generate the stacked spectra. The top spectrum shows a conventional zero-field NMR spectrum of the same sample, for reference. The observed oscillations are discussed in the main text and match the predictions from the simplified numerical simulations shown in Supplementary Figure 17.

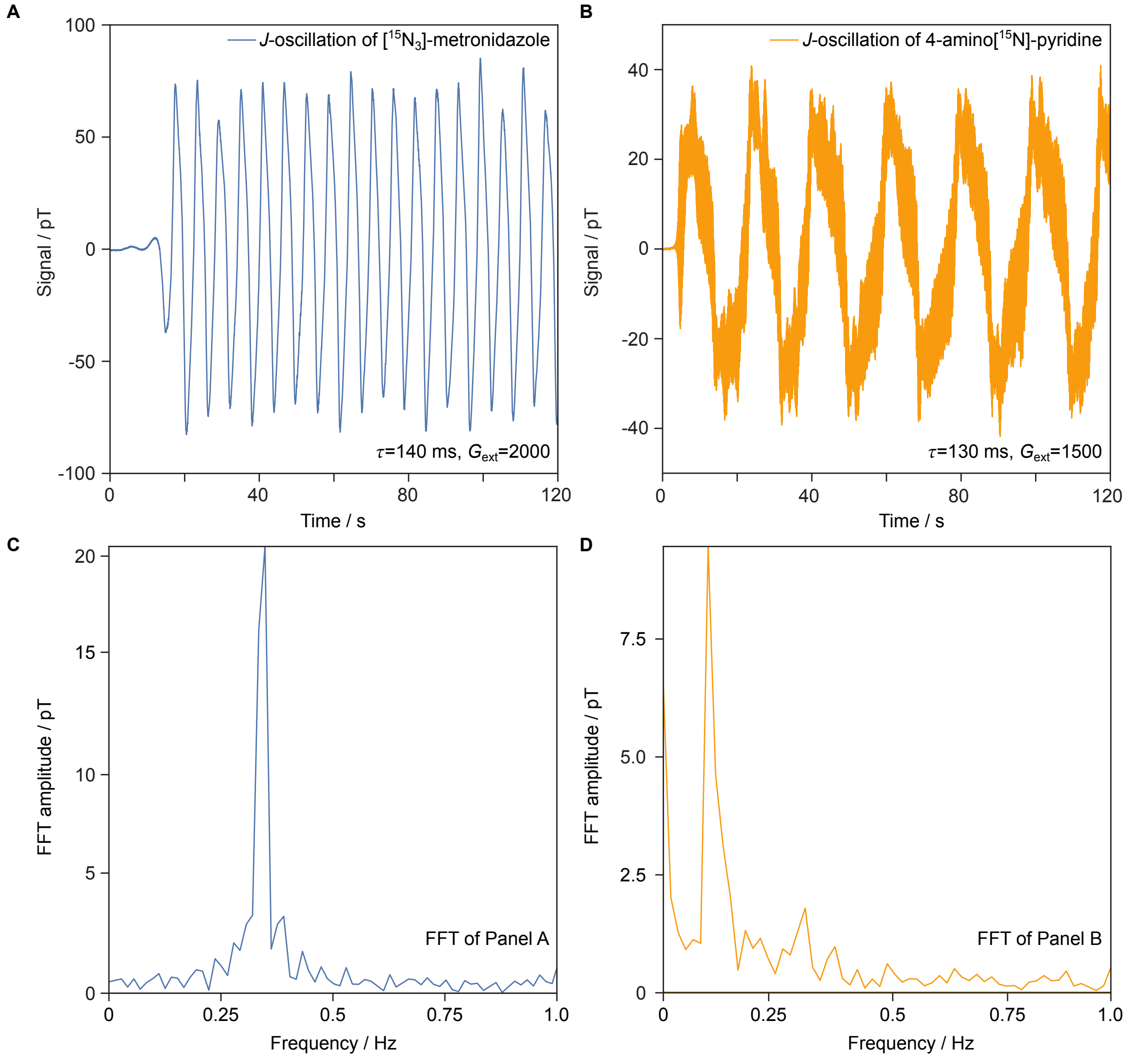

Supplementary Figure 7: **The** ***J*****-oscillators operating on heterocycle molecules generate ultralow frequency signals.** (A) $[^{15}N_3]$-metronidazole oscillator acquired with $G_{ext} = 2000$ and $\tau = 140\,\text{ms}$; (B) 4-amino$[^{15}N]$-pyridine oscillator acquired with $G_{ext} = 1500$ and $\tau = 130\,\text{ms}$. Panels (C) and (D) show the Fast Fourier Transform (FFT) of the oscillator signals from panels (A) and (B), respectively, computed over the 20–120 s time window.

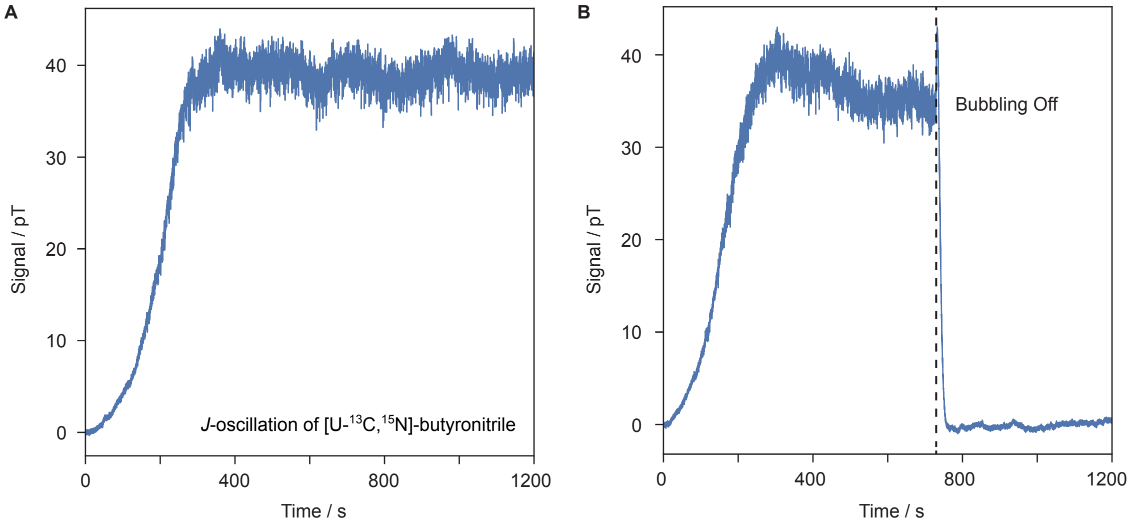

Supplementary Figure 8: The *J*-oscillator on [U-^{13}C, ^{15}N]-butyronitrile. The data were acquired with $G_{\mathrm{ext}} = -2000$ and $\tau = 150\,\mathrm{ms}$. (A) A 20-min acquisition produces a DC maser signal (no oscillations are resolvable within the acquisition window). (B) The signal acquired under the same conditions, but with *para*-H_2 bubbling stopped at around 700 s (marked as the dashed line). The signal disappears upon stopping the bubbling, confirming that its origin relates to sample hyperpolarization and feedback.

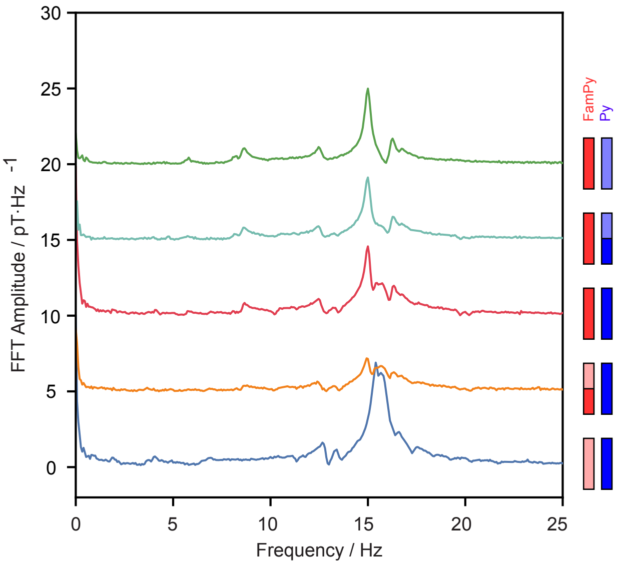

Supplementary Figure 9: Zero-field NMR spectrum of five pyridine / 4-aminopyridine mixtures tested in Fig. 5. The compositions of each sample are denoted by the same defined bar pairs as in Fig. 5, which indicate the specific ^{15}N isotopic enrichment of both pyridine and 4-aminopyridine substrates.

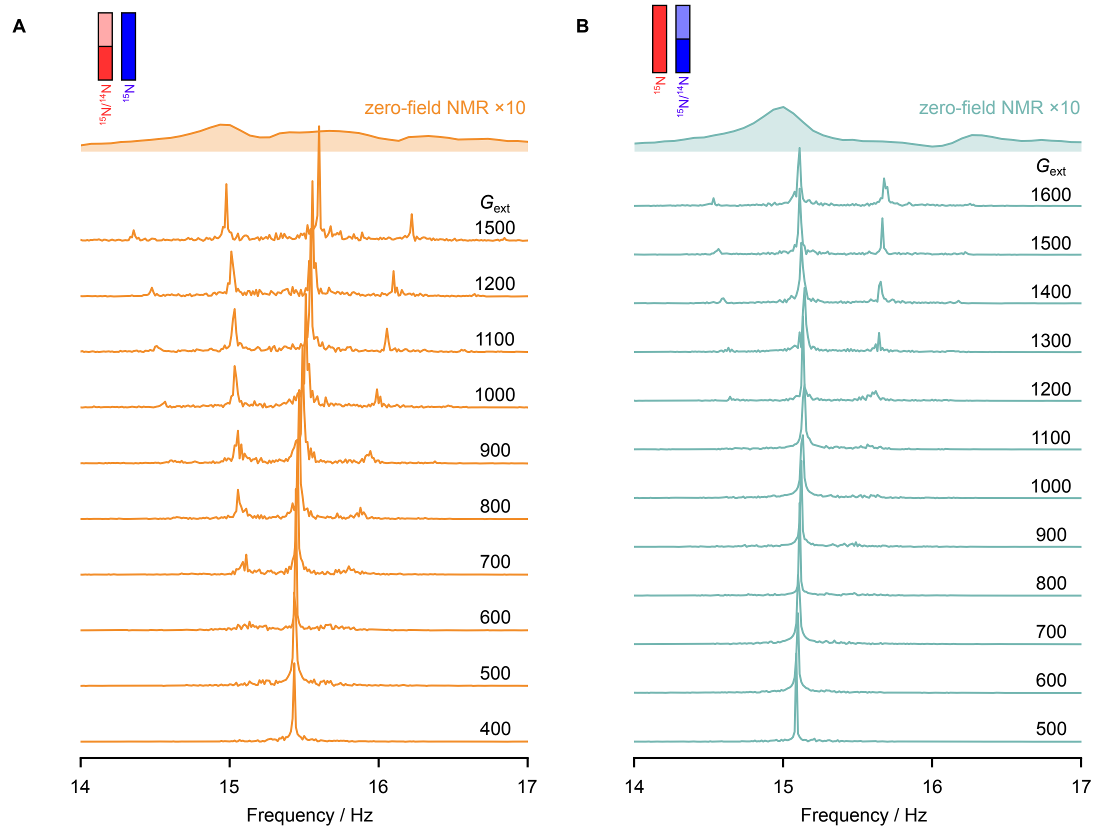

Supplementary Figure 10: **Quantum J-oscillators operated on pyridine / 4-aminopyridine mixture with different ^{14}N/^{15}N isotopic compositions.** The samples are denoted by the same bar pairs as in Fig. 5. (A) Sample with 100 % ^{15}N-enriched pyridine and 50 % ^{15}N-enriched 4-aminopyridine. (B) Sample with 50 % ^{15}N-enriched pyridine and 100 % ^{15}N-enriched 4-aminopyridine. The feedback delay is fixed at 115 ms for all measurements and the applied feedback gain is annotated to the left of each trace. For reference, the conventional zero-field nuclear magnetic resonance (NMR) spectrum of each sample are shown as shaded traces, scaled up by a factor of 10 for clarity.

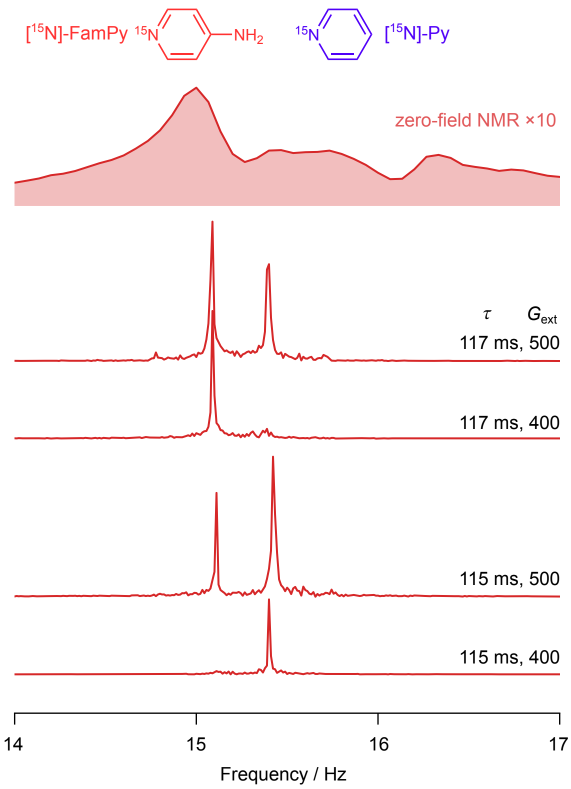

Supplementary Figure 11: ***J*-oscillators for mixtures of [^{15}N]-pyridine ([^{15}N]-Py) and 4-amino[^{15}N]-pyridine ([^{15}N]-FamPy) measured under the indicated feedback parameters.** For reference, the conventional zero-field nuclear magnetic resonance (NMR) spectrum of the sample are shown as shaded traces, scaled up by a factor of 10 for clarity.

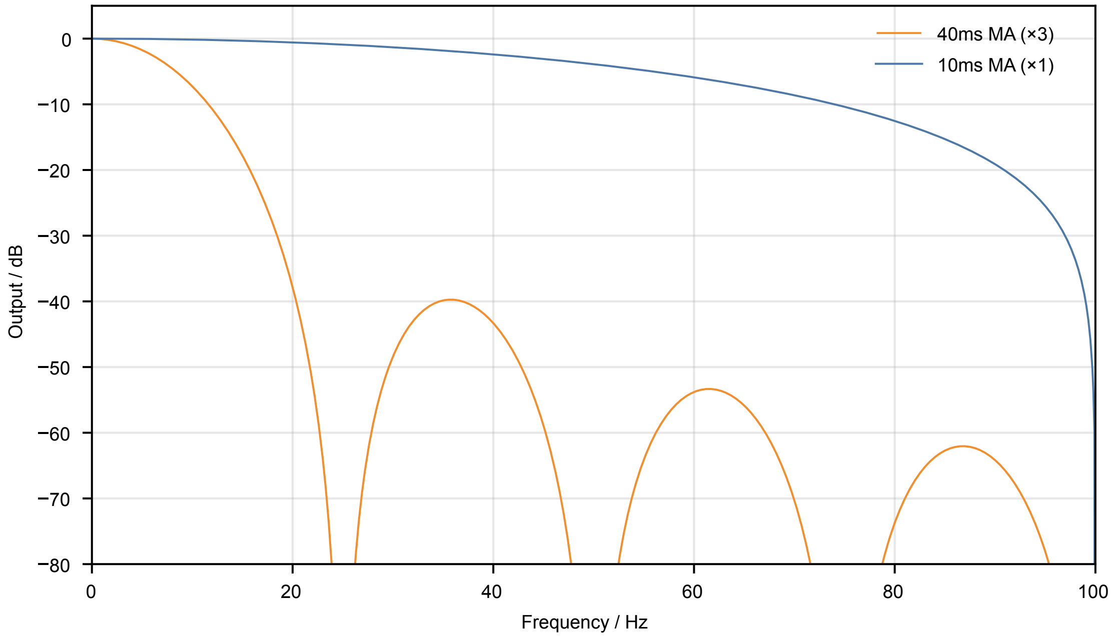

Supplementary Figure 12: **Amplitude Response of Moving-Average Filters.** A 40 ms moving-average filter cascaded three times (orange); a single 10 ms moving-average filter (blue). The amplitude responses were calculated numerically using standard signal-processing functions implemented in *SciPy*.

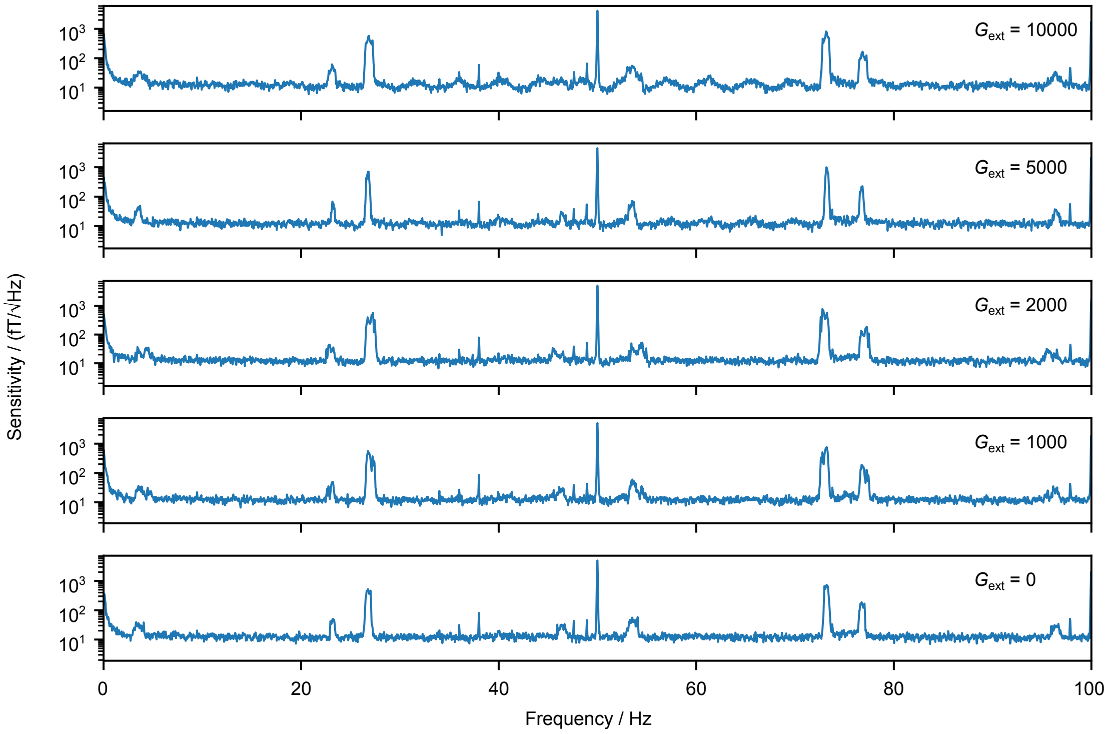

Supplementary Figure 13: **Stacked noise floors of the OPM sensor under varying external feedback gains (G_{ext}).** Measurements were performed without a sample, with the feedback delay fixed at τ=222 ms. A moving-average filter (10 ms window size, applied once) was included in the feedback loop. Each noise floor was obtained from the amplitude spectral density calculated over a 30 s time-domain trace and averaged over 10 repetitions.

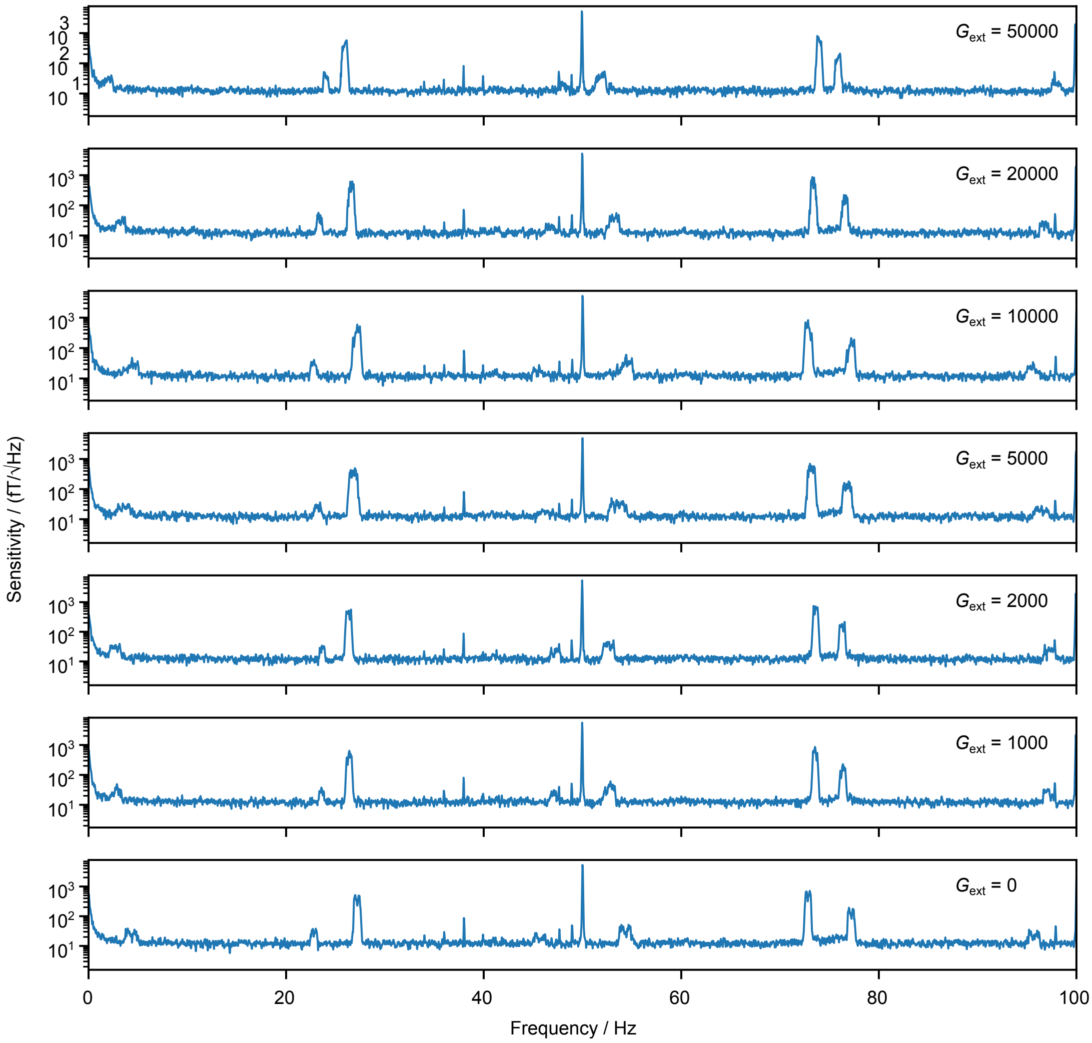

Supplementary Figure 14: Stacked noise floors of the OPM sensor under varying external feedback gains (G_{ext}). Measurements were performed without a sample, with the feedback delay fixed at τ=222 ms. A moving-average filter (40 ms window size = 40 ms, applied three times) was included in the feedback loop. Each noise floor was obtained from the amplitude spectral density calculated over a 30 s time-domain trace and averaged over 10 repetitions.

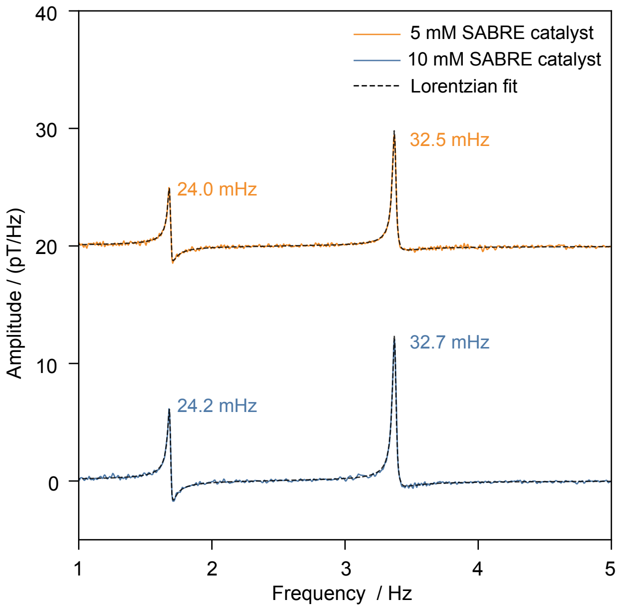

Supplementary Figure 15: **Dependence of the signal linewidths on the catalyst concentrations.** Zero-field spectra of naturally abundant [^{15}N]-ACN / [^{14}N]-ACN samples (0.36 % / 99.6 %) with catalyst concentrations of 5 mM (orange) and 10 mM (blue). The co-ligand (benzylamine) to catalyst concentration ratio was 25 for both samples. Dashed lines closely following the experimental data represent dual Lorentzian fits, from which the full-width-at-half-maximum of the corresponding peaks was extracted.

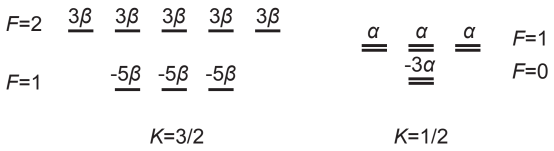

Supplementary Figure 16: **Visualization of $\hat{\rho}_{\mathrm{eq}}$ at zero-field.** The parameters α and β (with coefficients) denote the offsets of populations with respect to the thermal state at zero-field. The states with the same total proton angular momentum K and total angular momentum F have identical populations.

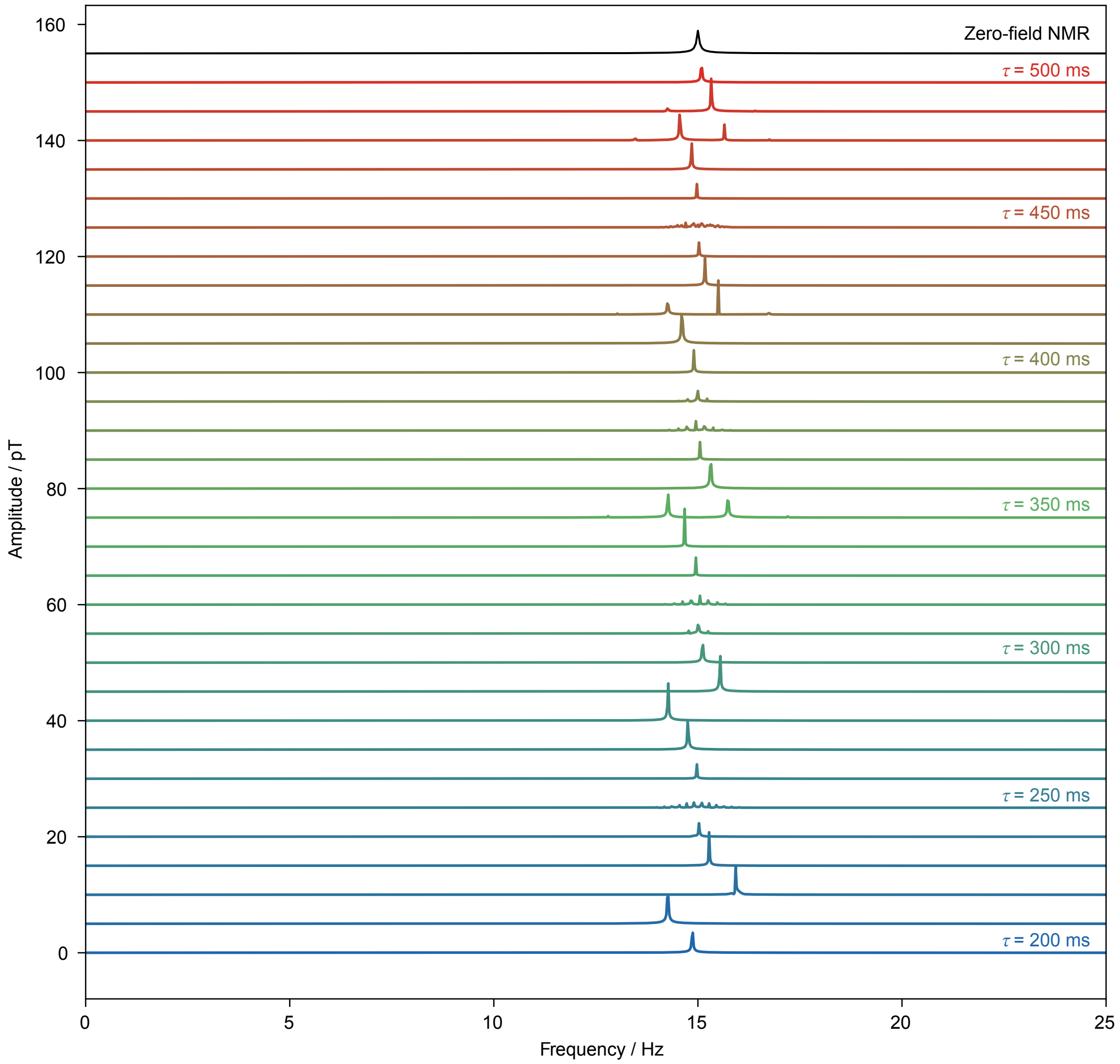

Supplementary Figure 17: **Simulations of J-oscillators operating on coupled ^{15}N-^{1}H two spin system.** Simulations assume a scalar J coupling constant of 15 Hz, OPM rms noise of 0.1 pT and identical system geometry to previous experiments. The SABRE-pumped population imbalances are set such that the integral of peak in the conventional zero-field NMR measurement equals to 50 pT. The nuclear spin relaxation are accounted using random fluctuating field model, resulting in resonance linewidths (FWHM) of 0.2 Hz in conventional J-spectra. The external feedback gain is fixed at $G_{\text{ext}} = -3000$ for all simulation. Each spectrum corresponds to a 1 min acquisition, with Fourier transformation applied to the time-domain data from 20–60 s to generate the stacked spectra. The top spectrum shows a simulation of conventional zero-field NMR spectrum of the system, for reference. Simulations were performed following the Methods Section.

Supplementary Tables

Supplementary Table 1: External feedback phase lags at oscillator frequencies for given delay intervals. The delay intervals show where the quantum oscillator on [^{15}N]-ACN is sustained, as extracted from Figure 3. Parentheses indicate the initial or end sampling intervals rather than real cutoffs. The phase lag is derived using $\varphi = 2\pi f\tau$ at around the J-transition frequencies $f = J$ or $f = 2J$ ($J = 1.687$ Hz), accounting for frequency-dependent phase lags due to delay. For the negative external feedback gain ($G_{\text{ext}} = -20$), an additional π phase lag is added.

G_{ext}	Oscillator	Delay (ms)	Phase Lag φ (rad)
-20	1-J	$\langle 60\rangle$–275	$\langle 3.78\rangle$–6.05
	2-J	$\langle 60\rangle$–140	$\langle 4.41\rangle$–6.11
20	1-J	325–$\langle 400\rangle$	3.44–$\langle 4.24\rangle$
	2-J	160–290	3.39–6.14

Supplementary Table 2: **Sample compositions for *J*-oscillators; model (Figs. 1-2) and molecules A-I (Fig. 3); PTC = polarization transfer catalyst.**

**Sample**	**Substrate**	**PTC [mM]**	**Co-substrate**	**Solvent**
Model	$[^{15}N]$-ACN (5 %)	5.0	$BnNH_2$ (125.0 mM)	ACN
A	$[^{15}N]$-ACN (0.36 %)	5.0	$BnNH_2$ (125.0 mM)	ACN
B	$[1\text{-}^{13}C,^{15}N]$-ACN (1.0 %)	5.3	$BnNH_2$ (125.0 mM)	ACN
C	$[2\text{-}^{13}C,^{15}N]$-ACN (1.0 %)	5.1	$BnNH_2$ (125.0 mM)	ACN
D	$[^{15}N]$-pyridine (100.0 mM)	5.0	-	MeOH
E	$[^{15}N_2]$-imidazole (97.1 mM)	4.9	-	MeOH
F	$[^{15}N_3]$-metronidazole (53.5 mM)	4.2	-	MeOH-d_4
G	4-amino$[^{15}N]$-pyridine (92.9 mM)	4.4	-	MeOH
H	$[1\text{-}^{13}C]$-pyruvate (76.8 mM)	6.3	DMSO (24.1 mM)	MeOH
I	$[U\text{-}^{13}C, ^{15}N]$-butyronitrile (107.4 mM)	5.5	pyridine (52.4 mM)	MeOH/MeOH-d_4 (1:1)

Supplementary References

[1] Xu, J., Kircher, R., Picazo-Frutos, R., Budker, D., Barskiy, D.: Zero-to ultralow-field control of hyperpolarized nuclear spin orders in acetonitrile solvent (2025). 10.26434/chemrxiv-2025-h53bc

[2] Levitt, M.H.: Demagnetization field effects in two-dimensional solution NMR. Concepts Magn. Reson. **8**(2), 77–103 (1996)

[3] Xu, J., Barskiy, D.A.: Essential tools of linear algebra for calculating nuclear spin dynamics of chemically exchanging systems. J. Magn. Reson. Open **16**, 100132 (2023)

[4] Strang, G.: On the construction and comparison of difference schemes. SIAM J. Numer. Anal. **5**(3), 506–517 (1968)
