# Supplementary material for: Quantum magnetic J-oscillators
Source: Nat Commun. 2026 Jan 29;17:1200. doi: 10.1038/s41467-026-68779-5 (PMC12859129; doi:10.1038/s41467-026-68779-5)
Supplement: Supplementary file 2 — Description of Additional Supplementary Information [file 41467_2026_68779_MOESM2_ESM.pdf]

Description of Additional Supplementary Files

File Name: Supplementary Software 1
Description: The feedback loop is implemented in the main.py script. Prior to execution, the user must ensure that the device and channel identifiers (Dev, AI_CHANNEL, AO_CHANNEL) correspond to the installed NI-DAQ hardware, that the serial port (bub_port) is correctly specified for the host platform, and that the output directory (save_dir) exists and is writable. The script relies on functions defined in oscillator_manager.py, which provide utilities for cyclic buffer read/write operations, HDF5 data storage, timer-based control of the acquisition duration, and thread-safe status reporting during runtime. All free parameters of the experiment are defined in the Parameters block of the code and are described in detail in the Methods section. Further details of the feedback algorithm are provided in the main manuscript under the Methods section.
